# Redefining Death: Urgent Need to Evolve toward a Homogeneous Definition of Death in India

**DOI:** 10.5005/jp-journals-10071-23220

**Published:** 2019-08

**Authors:** Sonali Vadi, Sunil Shroff

**Affiliations:** 1 Department of Intensive Care Unit, Kokilaben Dhirubhai Ambani Hospital and Medical Research Center, Mumbai, Maharashtra, India; 2 Department of Intensive Care Unit, MOHAN Foundation, Chennai, Tamil Nadu, India

**Keywords:** Brainstem death, Circulatory-respiratory criteria for death, Defining death, Ethical issues, Legal issues

## Abstract

The interdependent circulatory-respiratory criteria and brainstem criteria are used to define death. Continuing life-support in brainstem dead patients is a legal conundrum for the health-care professionals and an ethical worry for the society at large. There is an urgent need to revisit this subject to evolve a more uniform definition of death irrespective of the circumstances. A compelling take-home message from this discussion is to spread awareness about brainstem death amongst healthcare professionals

**How to cite this article:**

Vadi S, Shroff S. Redefining Death: Urgent Need to Evolve Toward a Homogeneous Definition of Death in India. Indian J Crit Care Med 2019;23(8):368–370.

## INTRODUCTION

Circulation, respiration and brain function are all independently vital to life.

Brainstem death denotes an irreversible loss of capacity for consciousness (injury to reticular activating system) and irreversible loss of the capacity to breathe (injury to nuclei for cardiorespiratory regulation).^1^ Brainstem death determination requires going along with laid down neurological criteria for its determination. The current standards and minimum clinical criteria to determine such death vary from country to country, and within a country from one hospital to another. These inconsistencies lead to confusion in concepts and dilemmas in documentation of the process. The varying standards for brain-death testing include the minimum required qualification of the examiner, the prerequisite blood pressure, temperature, the number of evaluations, and the requirement of ancillary tests like EEG or cerebral angiography. Other inconsistencies include the concept of brain death itself. For example, United States follows the concept of whole brain death while United Kingdom and India follows the concept of brainstem death. The recent recommendation by the World Brain Death Project is to abandon terms like whole brain death or brainstem death and adhere to determination of death by neurological criteria and remove these inconsistencies in practice.^2^

### Problems in India When Brain Death is Linked to Organ Donation

In India, brain death is linked to organ donation and is utilized for this purpose alone. On the other hand, acceptance of brain death to forgo ventilatory support remains in the want. Declaring brainstem death and stopping ventilatory support awaits inclusion into ‘accepted medical practice’. In a situation of brainstem death where family does not wish to opt for organ donation, intensivists in many hospitals are unsure if ventilatory support can be discontinued. The uncertainty results in unnecessary ventilation and prolongs the ICU stay. Discrepancies in the definitions of death and tagging brainstem death with organ donation leads to ethical and legal issues at the bedside.^3^

### Regulations Governing Death in India

Death is a part of three different laws in India, viz. The Indian Penal Code (IPC, 1862), The Registration of Births and Deaths Act (RBDA, 1969), and Transplantation of Human Organs Act (THOA, 1994). These are not mutually inclusive

- In Section 46 of IPC—Death is defined as ‘death of a human being unless contrary appears from the context’.
- In Section 2(b) of RBDA—Death is ‘the permanent disappearance of all evidence of life at any time after live-birth has taken place.’ ^4^
- In Section 2(e) of THOA— Death is ‘the permanent disappearance of all evidence of life by reason of brainstem death or in a cardiopulmonary sense at any time after live birth has taken’.^5^

Can we medically and legally accept that in brain death there is ‘permanent disappearance of all evidence of life,’ as the heart is still beating, and the body is somatically functional. In fact, there are instances in the literature where birth of normal healthy baby has happened from brain dead mothers.^6^

In India with no ‘one’ uniform definition of death the reference point of death can be as per any of the three laws and can be interpreted differently. Evidently, brainstem death equates to legal death only in THOA. Similarly, dead donor rule is not consistent with circulatory death for heart transplantation,^7^ controlled and uncontrolled as per the present law, requires redefinition. India has a death rate of 7.3 per 1000 population annually.^8^ This translates to approximately 26,700 daily deaths. Deaths occur due to various causes. It is estimated that of these deaths, brain death happens in approximately 250 individuals everyday due to road traffic accidents alone.^9^ Of these approximately 2.5–3 go on to donate organs and the large majority are neither identified or certified. With improving intensive care there are likely to be improvement in this rate of identification. Consequentially with time even more intensivists will face the dilemma as organ donation has becomes a regulatory part of end of life care discussion with the family as per the 2011 amended THOA law.^10^ The ‘required request’ clause in the amended law makes it compulsory for ICU clinicians to provide an organ donation option to the family with the help of the transplant coordinator in the registered transplant hospitals, and non-transplant hospitals that have an ICU

Death certificate is available in two different formats in India: The Registration of Births and Deaths Act (1969) and Transplantation of Human Organs Rules (1995- brainstem death certificates Forms 8 and 10).^11^ However, the THOA brain death certificate has no locus standi for the cremation or for insurance claim. This certificate is used only when organ donation takes place. This means brain death as cause of death will never be officially acceptable and its true incident in the country will never be known. This anomaly too requires clarification from the regulatory authorities.

To overcome all the above confusion, we require to make the definition of death uniform in India.

### Dignity in Death

Brainstem death equates to legal death as per THOA notably for the purposes of organ donation. Thus, withdrawal of life support is linked to it. The downstream effect of this intention introduced into the definition of death is its objectionable consequences. Four aspects to this:

1. Post-second positive apnea test which is officially the time of death, there have been instances of family refusing on their decision to donate and ask that ventilatory support be discontinued;
2. Consequentially it fails to follow the mandatory calling for declaration of brainstem death as set by some state governments (e.g. Maharashtra,^12^ Tamil Nadu), especially in confrontational situations;
3. This would lead to unnecessary continuation of ventilatory support (no guidelines are in place on ventilator discontinuation in cases of refusal to organ donation), triage and engagement of human resources in maintaining a brainstem dead patient, and compromise moral integrity of treating doctor.^13^
4. If family needs time prior to consenting for organ donation and in the interim the deceased suffers cardio-pulmonary death, what should be the reporting time of death – should it be recorded as the time of declaring brainstem death or after circulatory death.^14^

Additionally, continuing life-support after declaration of brainstem death (which is equivalent to legal death in India) may lead family members as well as healthcare providers to doubt the lawful status of a patient. In the least that any healthcare professional can do as a part of end-of-life care is to ensure dignity of a patient in death.^15^ Legal ambiguity adds to the emotional stress of any family. This also creates mistrust on the motives of treating doctors. Escape from this impasse for ethical and legal safety of the health care system can be realized by de-coupling brainstem death from organ donation.

### Redefining Death

Measures required to help follow the best practices requires the following:

- Increasing healthcare professional's education toward improving their attitudes and perceptions to brainstem death,
- Providing them with best practice training on diagnosis and interpretation of brainstem death testing
- Training of healthcare professional in breaking the bad news and broaching on the subject of organ donation
- Integrating the best practices into in the end of life family conversation.

Legal obligations that are associated with determination and declaration of death by cardiopulmonary criteria should apply to determination and declaration of brainstem death also. Given that we have a formalized brain death protocol, a need for more uniform definition of death,^3^ one that encompasses brainstem death and circulatory death is desirable. A homogeneous definition would further serve as a prelude toward more efficient transplant network. Clinical diagnosis of brain death cannot confirm irreversible loss of all intracranial neurological functions.^16^

## CONCLUSION

Neurological criteria-based determination of death though accepted at present is a means to an end for the purposes of organ donation leading to controversy between end-of-life care and organ donation. Discussions on organ donation should be a sequela of brain death declaration rather than being a primary reason to declare brain-death as per the existing THOA regulation. A more unified definition of death would protect rights of patient and healthcare professionals thus reinforcing their trust in the regulations governing healthcare in India.
